# Creating high‐resistant starch rice by simultaneous editing of *SS3a* and *SS3b*

**DOI:** 10.1111/pbi.14053

**Published:** 2023-05-01

**Authors:** Lichun Huang, Ying Xiao, Wei Zhao, Yanan Rao, Huimin Shen, Zhengwen Gu, Xiaolei Fan, Qianfeng Li, Changquan Zhang, Qiaoquan Liu

**Affiliations:** ^1^ Jiangsu Key Laboratory of Crop Genomics and Molecular Breeding, Key Laboratory of Plant Functional Genomics of the Ministry of Education, College of Agriculture Yangzhou University Yangzhou China; ^2^ Co‐Innovation Center for Modern Production Technology of Grain Crops of Jiangsu Province, Jiangsu Key Laboratory of Crop Genetics and Physiology Yangzhou University Yangzhou China

**Keywords:** *Oryza sativa* L., resistant starch, soluble starch synthase, *SS3a*, *SS3b*

The conventional starch from cereals is usually high in energy and easily digested, absorbed and converted into blood sugar in human small intestine, while resistant starch (RS) is hardly enzymatically hydrolysed into glucose in human small intestine and only fermented into beneficial short‐chain fatty acids in large intestine (Jukanti *et al*., 2020; Shen *et al*., 2022). Thus, RS‐rich foods can not only effectively reduce the glycaemic index, increase satiety and prevent blood sugar‐related diseases but also contribute to the prevention of intestinal‐related diseases by improving the intestinal micro‐environment and decreasing colonic pH (Zaman and Sarbini, 2016). Rice is an important staple‐food crop and a major source of starch for majority of the population, especially in Asia. However, the RS content in conventionally cultivated rice is usually <1%, which is far below the daily recommended RS intake for humans (Yadav *et al*., 2010). Therefore, breeding rice rich in RS is an important target for rice variety improvement.

Rice endosperm is the chemical reservoir storing more than 80% starch with varied amounts of amylose and amylopectin, which is mainly controlled by several key starch synthase‐related genes (SSRGs; Huang *et al*., 2021). Currently, there have been a few successful cases of altering the composition and structure of starch to enhance RS content in rice endosperm by modifying the expression of two kinds of SSRGs, *SBE* and *SS3*/*SSIII*, encoding starch branching enzyme and soluble starch synthase III, respectively (Butardo Jr. *et al*., 2012; Guo *et al*., 2020; Miura *et al*., 2022; Zhou *et al*., 2016; Zhu *et al*., 2012). Loss of function of *SBE3/SBEIIb* resulted in increased RS in rice endosperm, while there is no significant effect of *SBE1/SBEI* mutation on RS level, but the *sbe1sbe3* double mutation could further enhance RS content substantially based on *sbe3* single mutation, indicating functional redundancy of these two isoforms in RS formation (Zhu *et al*., 2012). In rice, there are two isoforms of SS3/SSIII, SS3a/SSIIIa/SSIII‐2 and SS3b/SSIIIb/SSIII‐1 (Huang *et al*., 2021). Zhou *et al*. (2016) reported that the *ssIIIa/ss3a* loss‐of‐function mutant had a high RS content in cooked rice. However, the role and genetic effects of most other SSRGs, such as *SS3b*, in the formation of RS are not clear.

In rice, *SS3a* and *SS3b*, two genes encoding the SS3 isoforms, and their gene structure, protein domain and amino acid sequence were quite conserved (Figure S1a–c; Table S2). *SS3b* was expressed in low abundance in developing rice grains, but its expression trend was consistent with that of *SS3a* (Figure S1d,e). It implied that *SS3b* may also have a similar function as *SS3a* in regulating starch synthesis and RS formation in rice endosperm. Thus, we created *SS3a* and *SS3b* mutants by CRISPR/Cas9 genome editing technology in the *japonica* rice cultivar Nipponbare (WT). After several generations of genotype and phenotype screening, we obtained several homozygous mutant lines, including *SS3a* single mutants (*ss3a*), *SS3b* single mutants (*ss3b*) and their pyramiding double mutant lines (*ss3a*‐*ss3b*) (Figure 1a,b). There was no significant changes in the seedling growth and plant development of all edited rice lines compared with their wild type (WT; Figure S2a,b).

**Figure 1:**
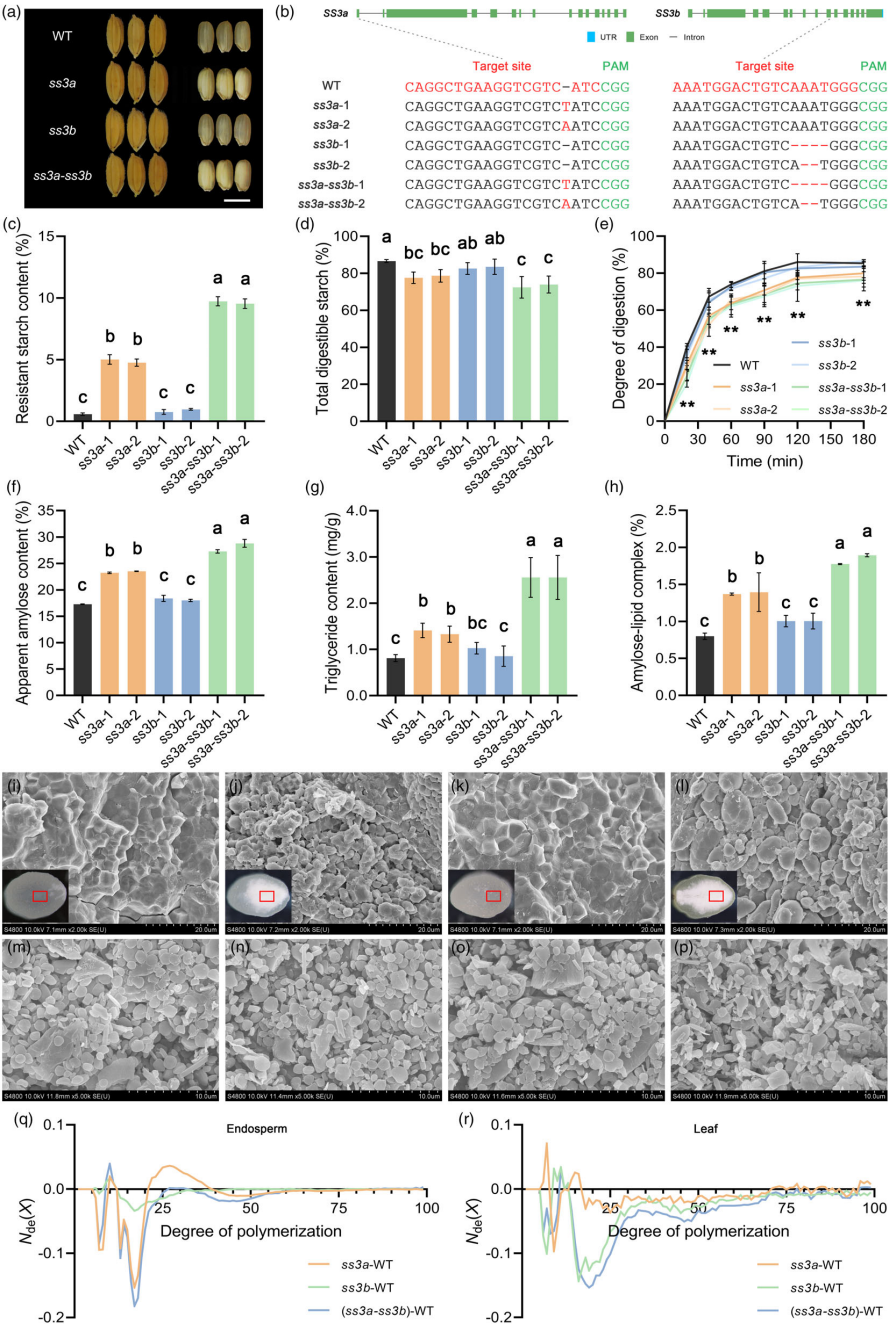
Generation and characteristics of *ss3* mutants and their wild type (WT). (a) Grain morphology. Scale bar = 5 mm. (b) The schematic diagram of target sites and mutation sites. Font marked in red indicates the target site or the changed base sequence. (c, d) The contents of and in rice flour. (e) *In vitro* digestion rate of cooked rice. (f–h) The contents of apparent amylose (f), triglyceride (g) and amylose–lipid complex (h) of rice flour. (i–p) Starch granule morphology of grain cross section (i–l) and purified leaf starches (m–p). From left to right are WT, *ss3a*, *ss3b* and *ss3a*‐*ss3b* mutants, respectively. The red box (i–l) represents the viewing area of the SEM. The observation multiples are 2000 (i–l) and 5000 (m–p), respectively. (q, r) Amylopectin chain‐length distribution of purified starches from endosperm (q) and leaves (r). Different lower‐case letters indicate statistically significant differences at *P* < 0.05, *n* = 3. “**” indicates statistically significant differences between *ss3a*, *ss3a*‐*ss3b* mutants and WT at the *P* < 0.01 level, *n* = 3.

The digestive characteristics of the grains from the above mutants were firstly determined. The results showed that the RS content in *ss3a* mutants increased significantly to 4.76%–5.01% while that in WT was only 0.58% (Figure 1c). Moreover, the total digestible starch (TDS) content and digestion rate in *ss3a* mutants decreased significantly (Figure 1d,e), which was consistent with previous report (Zhou *et al*., 2016). In *ss3b* mutants, there was no significant change in the contents of RS and TDS as well as digestion rate compared with those in wild type. But *SS3b* mutation could significantly enhance the RS level in the background of *SS3a* mutation, resulting in the RS content reaching 9.54%–9.73% in the *ss3a*‐*ss3b* double mutants, and further decreased TDS content and digestion rate (Figure 1d,e).

Except digestion properties, the *SS3b* mutation had no significant effect on other grain physicochemical properties, could further strengthen the change of grain physicochemical phenotype combined with the *SS3a* mutation (Figure 1f,g and Figure S3a–c; Tables S3 and S4). These results suggested that *SS3b* can only have a significant effect on starch synthesis and RS formation in rice endosperm based on *SS3a* mutation, indicating *ss3b* mutant has synergistic effects on *ss3a* mutant in increasing RS content.

The starch granule morphology of *ss3a* and *ss3a*‐*ss3b* mutants was obviously abnormal, showing most compound starch grains composed of only a few starch granules, instead, dozens of starch granules formed a compound starch grain in the wild type (Huang *et al*., 2021), and some starch granules were spherical (Figure 1i–l and Figure S3e–h). Furthermore, the starch granules in each compound starch granule complex were closely linked, but loosely arranged between the adjacent compound starch granules in *ss3a* and *ss3a*‐*ss3b* mutants (Figure 1i–l). These results explained the high chalkiness phenotype in *ss3a* and *ss3a*‐*ss3b* mutants. The starch granule morphology of *ss3b* mutant had no obvious change compared with the wild type.

As to starch fine structure, the true amylose fraction (Am) increased significantly and the proportion of amylopectin long chains (Ap2) decreased dramatically in *ss3a* and *ss3a*‐*ss3b* mutants, and the range of changes in *ss3a*‐*ss3b* mutants was larger than that in *ss3a* mutants, but no significant change occurred in *ss3b* mutants (Figure S5a). Besides, the amylopectin B_2_ chains with degree of polymerization (DP) 22–37 increased in *ss3a* mutants, while the amylopectin A chains with DP 6–9 and B_1_ chains with DP 13–21 decreased dramatically in *ss3a* and *ss3a*‐*ss3b* mutants (Figure 1q). The X‐ray diffraction (XRD) results showed that the starch relative crystallinity of *ss3a* and *ss3a*‐*ss3b* mutants was significantly reduced (Figure S3d). Correspondingly, the area ratio of the characteristic peak, which representing the amylose–lipid complex as well as the type 5 RS (RS5), was significantly increased in the *ss3a* and *ss3a*‐*ss3b* mutants, reaching 1.7 and 2.3 times that of the wild type, respectively, consistent with the changes in triglyceride and RS contents (Figure 1h). Expectedly, the *SS3b* mutation had no significant effect on the relative crystallinity and RS5 content of rice endosperm starch compared with the wild type (Figure 1h and Figure S3d). It implied that the increased contents of amylose and amylose–lipid complex are the main  reasons for the high RS content in *ss3a* and *ss3a*‐*ss3b* mutants.

For transitory starch in leaves, there was no significant change in starch composition, structure, and starch granule morphology after *SS3a* mutation, while in *ss3b* mutants, TSC and proportions of amylopectin B chains (DP > 12) were significantly decreased, AAC and true AC were slightly increased, and starch granules were irregular in shape and rough in surface, similar to the effect of *SS3a* mutation on endosperm starch synthesis (Figure 1m–r, Figures S4 and S5b). The TSC and proportions of amylopectin B chains in leaves of *ss3a*‐*ss3b* mutant were further decreased, and the abnormal morphology of starch granules was aggravated, but there was no significant change on AC (Figure 1m–r, Figures S4 and S5b). These results suggested that *SS3b* plays a major role in leaf starch synthesis and *ss3a* mutant has synergistic effects on *ss3b* mutant.

In conclusion, our data indicated that *SS3a* and *SS3b* have synergistic effects in starch biosynthesis. In rice endosperm, further mutation of *SS3b* could strengthen the effect of *SS3a* mutation on alteration of starch physicochemical properties and RS content. A new rice germplasm with significantly increased RS content and improved digestive properties was created by co‐knockout *SS3a* and *SS3b*. These results provide a new strategy to breed novel rice varieties with high RS content and further help to elucidate the functions of SSRGs in cereals.

## Conflict of interest

The authors declare no conflict of interest.

## Author contributions

Q.Q.L., L.H. and C.Z. conceived the project. L.H., Y.X., W.Z., Y.R., H.S., D.Z., X.F., C.Z. and Q.F.L. carried out the experiments and analysed the data. L.H. and Q.Q.L. wrote the manuscript. All authors read and approved of the manuscript.

## Supporting information


**Data S1** Supplementary Materials and Methods.
**Figure S1‐S5** Supplementary Figures
**Table S1‐S4** Supplementary Tables.
