# Supplementary material for: Creating high‐resistant starch rice by simultaneous editing of SS3a and SS3b
Source: Plant Biotechnol J. 2023 May 1;22(4):787–9. doi: 10.1111/pbi.14053 (PMC10955483; doi:10.1111/pbi.14053)
Supplement: Supplementary file 1 — Data S1 Supplementary Materials and Methods. Figure S1‐S5 Supplementary Figures Table S1‐S4 Supplementary Tables. [file PBI-22-787-s001.docx]

**Supplementary Text**

The *SS3b* mutation had no significant effect on other grain physicochemical properties, that is, they had comparative levels of total starch content (TSC), apparent amylose content (AAC), triglyceride content, gelatinization temperature (GT) and viscosity properties with its wild type (Figure 1f-g and S3a-c; Table S3 and S4). But, the *SS3b* mutation could further strengthen the change of grain physicochemical phenotype combined with the *SS3a* mutation, leading to further increase in AAC and triglyceride content, significant decrease in TSC, GT and RVA (Rapid Viscosity Analysis) profiles (Figure 1f-g and S3a-c; Table S3 and S4). These results suggested that *SS3b* can only have a significant effect on starch synthesis and RS formation in rice endosperm based on *SS3a* mutation, indicating *ss3b* mutant has synergistic effects on *ss3a* mutant in increasing RS content.

The rice grain length and width were similar among different edited lines and WT (Figure 1a and S2c-e), but the chalky grain percentage and chalkiness degree increased significantly in the cases of *SS3a* mutation, including *ss3a* and *ss3a*-*ss3b* mutant lines, and the 1000-grain weight decreased significantly due to the remarkable decrease of grain thickness (Figure S2f-i). Interestingly, there was no significant change in grain morphology and appearance of the *ss3b* mutants, but it exacerbated the negative effect of *ss3a* mutants on grain thickness, leading to further decrease in grain thickness and 1000-grain weight in *ss3a*-*ss3b* than that of the *ss3a* (Figure S2f-g).

**Supplementary Tables**

**Table S1.** The primers used in this study.

| Name of primer | Primer sequences (5'-3') | Usage |
| --- | --- | --- |
| pC1300-F | ACACTTTATGCTTCCGGCTC | Vector construction |
| T3 | ATTAACCCTCACTAAAGGGA |  |
| SS3a qRT-F | ATGCATGGTTTCAAGTTCAGAC | qRT-PCR |
| SS3a qRT-R | CTAGAAGAAGCGACTTCCGTAT |  |
| SS3b qRT-F | TGTTTTGGGTGGAAGAAGAGCA |  |
| SS3b qRT-R | GCTTTCCGTTCAGCACCGT |  |
| Actin01 qRT-F | CCAAGGCCAATCGTGAGAAGA |  |
| Actin01 qRT-R | AATCAGTGAGATCACGCCCAG |  |
| SS3a Cas-F | GGCACAGGCTGAAGGTCGTCATC | Target sites for CRISPR/Cas9 |
| SS3a Cas-R | AAACGATGACGACCTTCAGCCTG |  |
| SS3b Cas-F | GGCAAAATGGACTGTCAAATGGG |  |
| SS3b Cas-R | AAACCCCATTTGACAGTCCATTT |  |
| SS3a Test-F | GTCATCCGTGGGCATTTACCTT | Detection of Mutated sites |
| SS3a Test-R | CGAACGTGTAGTTGGCGTAG |  |
| SS3b Test-F | GCTTCCACCCTTCTCATACACA |  |
| SS3b Test-R | AACAGAACACGGCCAGGTCA |  |

**Table S2.** Amino acid sequence alignment of SS3a and SS3b isoforms in rice.

SS3a MEMALRPQSLLCPRSRLKVVIRPASSASGGGLAQYFLMTRRYTGSRIVRCMVSSSDCPNR

SS3b MEMAHSP---LCSRSRPVLVVRPATAATG--FAQPIIRCRRFTRTRLLRCLVASADYSKR

**** * ** *** * *** * * ** ** * * ** * * * *

SS3a KAKRTISLHTEVASSRGYAPRIAAESSIQEREHINSDEETFDTYNRLLRNESTEWKKL--

SS3b NPRRASTPKPKGAASRTYAPRPTVESSMKKIGQSGTDEGDLGTSNGKLSSEATEQTSNVE

* * ** **** *** ** * * * * **

SS3a DTTEVDLSQDVSSSSMRKVDATDEAKLDILEDDLPRNLLNGVTMGEVDMLDEAGAEDDVF

SS3b ESSEVDFSGNVSSSVF--LEGMDDAFEAETEEEVEQNQSPELSSESMDD-DAIDRKLDEY

*** * **** * * * * * * *

SS3a EVDLSALHNSTVGKMDAVNEVGTENDLFEVDLSALHSAAVGKVDVVDGAKAKEDLFEMDS

SS3b RGKISAL-----------------------------------------------------

***

SS3a LALHSVTMGKVDAINAAGAEGDKFEVDLSALASNNSMIEAVNVMDEAKAIEDTLEVDLSG

SS3b ------------------------------------------------------------

SS3a NATSSSTYGEVKFEVDSLGNTSSTVMYGPADGAYEPRSDEVTFKVDSSENASNNVMYGRA

SS3b ------------------------------------------------------------

SS3a DVVDESWADEGIFEVDFFTNASSGAEYGKVDVVDEAKTDDFTFEIDSLEKDSNNKMHGKA

SS3b ------------------------------------------------------------

SS3a HMVDEAWDDEAIFEVDLFGNASSIPIYGEVNVLDEARADDGKFEVDLLGNTSSNSTHEEV

SS3b ------------------------------------------------------------

SS3a DVVDEAQTGEATFEVDLLGNALSSAIYKEVPVMGGAQDDEVDVDFSINASITETEKEADA

SS3b ------------------------------------------------------------

SS3a VDEARVEDETFDMDLVGKQISIDSMNDDVVEEGTKHHRYPMLSSAFIEVKTIHETPVSLK

SS3b --------------------------------------------------------ISSK

* *

SS3a PELMSVVMDQEQDKPISSVYQQEGSIFNLHAENQSTVDFHEREQMAITFDKQKESVAKLS

SS3b PEPTSVSSTHVQD-----------------------------------------------

** ** **

SS3a KEDQQTAGLPEQNMSFDGVHRKSQSIIGLPFQHQSIVSSPEKYRSIVGFHGQNQSIISSH

SS3b -------------------------------------------RSIVGFHEQEKSVVSFH

******* * * * *

SS3a KQDKSIVGVPKKIQSIVGSTKHDDSIVGFRKQDRSIVSVPEQKQSIVGFHKQDLSIVAVS

SS3b EQDRSIVSVPEQSQPSSGVSGQNPT---------------EEKTIISG---QDVTEEA--

** *** ** * * * * * * ** *

SS3a EQNLSIVAIPRESQSKQISIVRRHDPLHLKEVETKDRDGISKKSGGDDDLPHMLFEEELS

SS3b ---------PEEITGKSI-----------------EREPLSRE-----------------

* * * * * *

SS3a QVEDVARAIAYKKQHEVDVISLTPDIQ-ESPQDNIDPQELRRMLQELADQNCSMGNKLFV

SS3b -----TEKVLFADDDPRIIKDEQYEPDIAPVQDDVDPQVLRRRLEELAEKNYLAGNKCFV

** *** *** * *** * *** **

SS3a FPEAVKANSTIDVYLNRNLSALANEPDVHIKGAFNSWRWRPFTERLHKSELSGDWWSCKL

SS3b FPEVVQADSVIDLYLNHSMSALASEPDILIKGAFNGWRWKKFTQKMHKSELTGDWWCCKL

*** * * * ** *** **** *** ****** *** ** ***** **** ***

SS3a HIPKEAYRLDFVFFNGRLVYDNNDSNDFVLQVESTMDEDSFEEFLVEEKKRELERVATEE

SS3b HIPKQAYRLDFVFFNGDTIYENNNHNDFVLQIESEINEHSFEDFLVEEKQRELERLAAEE

**** *********** * ** ****** ** * *** ****** ***** * **

SS3a AERRRHAEEQQRMGEQRAAEQAAREQAKKEIELKKNKLQNLLSSARTHVDNLWHIEPSTY

SS3b AERKRQAEEERRKEEERAAMEADRAQAKAEVEMNKNKLQNLLNSASRYADNLWYIEPHTY

*** * *** * * *** * * *** * * ******** ** **** *** **

SS3a RQGDTVRLYYNRNSRPLMHSTEIWMHGGCNSWTDGLSIVERLVECDDENGDWWYANVHIP

SS3b KAGDRVKLFYNRSSRPLMHNTEIWMHGGYNNWSDGLSIAEKLIKSYEKDGDWWYADVTLP

** * * *** ****** ******** * * ***** * * ****** * *

SS3a EKAFVLDWVFADGPPGNARNYDNNGRQDFHAILPNAMTNEEYWVEEENCIYTRLLHEIRE

SS3b EGALVLDWVFADGPPGNARNYDNNGRQDFHAVVPNNISEDLFWVEEEHMIFKRLQKERKE

* * *************************** ** ***** * ** * *

SS3a REEAIKIKVEKRAKMKSEMKEKTMRMFLLSQKHIVYTEPLEIRAGTTVDVLYNPSNTVLN

SS3b REDADRRKSEITAKMKAEMKEKTMRDFLLSQKHIVYTEPLEVRAGTTVDVLYNPSNTVLN

** * * * **** ******** *************** ******************

SS3a GKPEVWFRWSFNRWMHPSGVLPPKKMVKTEDGCHLKATVSVPSDAYMMDFVFSESEEGGI

SS3b GKPEVWFRCSFNRWTHPSGPLPPQKMVNAENGSHLRATVRVPLDAYMMDFVFSESEEGGI

******** ***** **** *** *** * * ** *** ** *****************

SS3a YDNRNGTDYHIPVSGSNAKEPPIHIVHIAVEMAPIAKVGGLADVVTSLSRAIQELGHHVE

SS3b YDNRNGMDYHSPVTDSVAKEPPMHIVHIAVEMAPIAKVGGLGDVVTSLSRAVQDLGHNVE

****** *** ** * ***** ****************** ********* * *** **

SS3a VILPKYNFMNQSNVKNLHVRQSFSLGGTEIKVWFGLVEDLSVYFLEPQNGMFGGGWVYGG

SS3b VILPKYDCLNLSNVKDLHYRQSFTWGNTEIKVWFGKVEDVPVYFLEPQNGMFWVGCVYGR

****** * **** ** **** * ******** *** *********** * ***

SS3a NDAGRFGLFCQSALEFLLQSGSSPHIIHCHDWSSAPVAWLYKEHYAESRLATARIIFTIH

SS3b NDESRFGFFCHSALEFLRQNGSSPDIIHCHDWSSAPVAWLFKEQYAQNGLSNGRVVFTIH

** *** ** ****** * **** *************** ** ** * * ****

SS3a NLEFGAHFIGKAMTYCDKATTVSHTYSKEVAGHGAIAPHRGKFYGILNGIDPDIWDPYTD

SS3b NLEFGAHHIGKAMARCDKATTVSYTYSREVSGHGAIAPHFSKFHGIRNGIDPDIWDPYSD

******* ***** ******** *** ** ******** ** ** *********** *

SS3a NFIPMHYTSENVVEGKNAAKRALQQRFGLQQTDVPIVGIITRLTAQKGIHLIKHALHRTL

SS3b NFIPVHYTSENVVEGKSAAKKALQQRLGLQQTDTPVVGIISRLTVQKGIHLIKHAIYRTL

**** *********** *** ***** ****** * **** *** ********** ***

SS3a ERNGQVVLLGSAPDPRIQSDFCRLADSLHGENHGRVRLCLTYDEPLSHLIYAGSDFILVP

SS3b ERNGQVVLLGSAPDHRIQGDFTNLASKLHGEYHGRVKLCLTYDEPLSHLIYAGADFILVP

************** *** ** ** **** **** **************** ******

SS3a SIFEPCGLTQLVAMRYGSIPIVRKTGGLYDTVFDVDHDKDRARVLGLEPNGFSFDGADCN

SS3b SMFEPCGLTQLTAMRYGSIPIVRKTGGLYDTVFDVDDDKDRAREQGLEPNGFSFEGADSN

* ********* ************************ ****** ********* *** *

SS3a GVDYALNR--------------------QQSLLGLKPAVGSTP---SAKGSWSKTGPGTG

SS3b GVDYALDRAITTWYDARDWFHSLCKRVMEQDWTWNRPALDYMELYHSARKN---------

****** * * ** **

SS3a LPWTTLNCTIQLTNFEAPIQRWQEKASIGRYYKLNETWLKVKIFYLSCRYKLTQTWFKVK

SS3b ------------------------------------------------------------

SS3a IFYLSYTYICRIKTLYSMHKQLWEYVSAMFPILSFNYEYLI

SS3b -----------------------------------------

By CLUSTAL W (1.83) multiple sequence alignment

**Table S3.** The gelatinization parameters determined by DSC of rice flours from different mutants and their wild type (WT).

| Sample | ΔH (J/g) | T_o_ (°C) | T_p_ (°C) | T_e_ (°C) |
| --- | --- | --- | --- | --- |
| WT | 7.74±0.16 a | 65.80±0.10 a | 71.47±0.06 a | 79.23±0.32 a |
| *ss3a*-1 | 5.72±0.33 cd | 59.30±0.53 c | 66.37±0.90 c | 73.93±0.85 bc |
| *ss3a*-2 | 6.43±0.75 bc | 59.47±0.15 c | 66.83±0.49 c | 74.80±0.95 b |
| *ss3b*-1 | 6.97±0.47 b | 64.97±0.12 ab | 70.27±0.12 b | 78.17±0.55 a |
| *ss3b*-2 | 6.78±0.50 b | 64.53±1.26 b | 70.07±0.25 b | 78.50±0.53 a |
| *ss3a*-*ss3b*-1 | 5.00±0.43 de | 57.47±0.25 d | 65.50±0.17 d | 73.30±0.80 c |
| *ss3a*-*ss3b*-2 | 4.35±0.59 e | 58.40±0.30 cd | 65.17±0.51 d | 74.27±0.83 bc |

All data are means ± standard deviations, n=3. Different lower-case letters indicate statistically significant differences at *P* < 0.05. T_p_, peak temperature; T_o_, onset temperature; T_e_, end temperature.

**Table S4.** The starch viscosity parameters measured by RVA of rice flours from different mutants and their wild type (WT).

| Samples | Peak Viscosity (cp) | Hot Paste Viscosity (cp) | Breakdown (cp) | Cool Paste Viscosity (cp) | Setback (cp) | Peak Time (min) | Pasting Temperature (℃) |
| --- | --- | --- | --- | --- | --- | --- | --- |
| WT | 4579 | 2764 | 1815 | 4221 | -358 | 6.0 | 79.0 |
| *ss3a*-1 | 1453 | 1150 | 303 | 1708 | 255 | 6.6 | 93.5 |
| *ss3a*-2 | 1259 | 1068 | 191 | 1667 | 408 | 6.8 | 94.3 |
| *ss3b*-1 | 4412 | 2840 | 1572 | 4239 | -173 | 6.3 | 75.7 |
| *ss3b*-2 | 4434 | 2741 | 1693 | 4196 | -238 | 6.1 | 75.7 |
| *ss3a*-*ss3b*-1 | 369 | 274 | 95 | 448 | 79 | 6.3 | > 95.0 |
| *ss3a*-*ss3b*-2 | 324 | 249 | 75 | 382 | 58 | 6.4 | > 95.0 |

Due to the limitation of rice flour, only one replicate was performed for each line.

**Supplementary Figures**

**
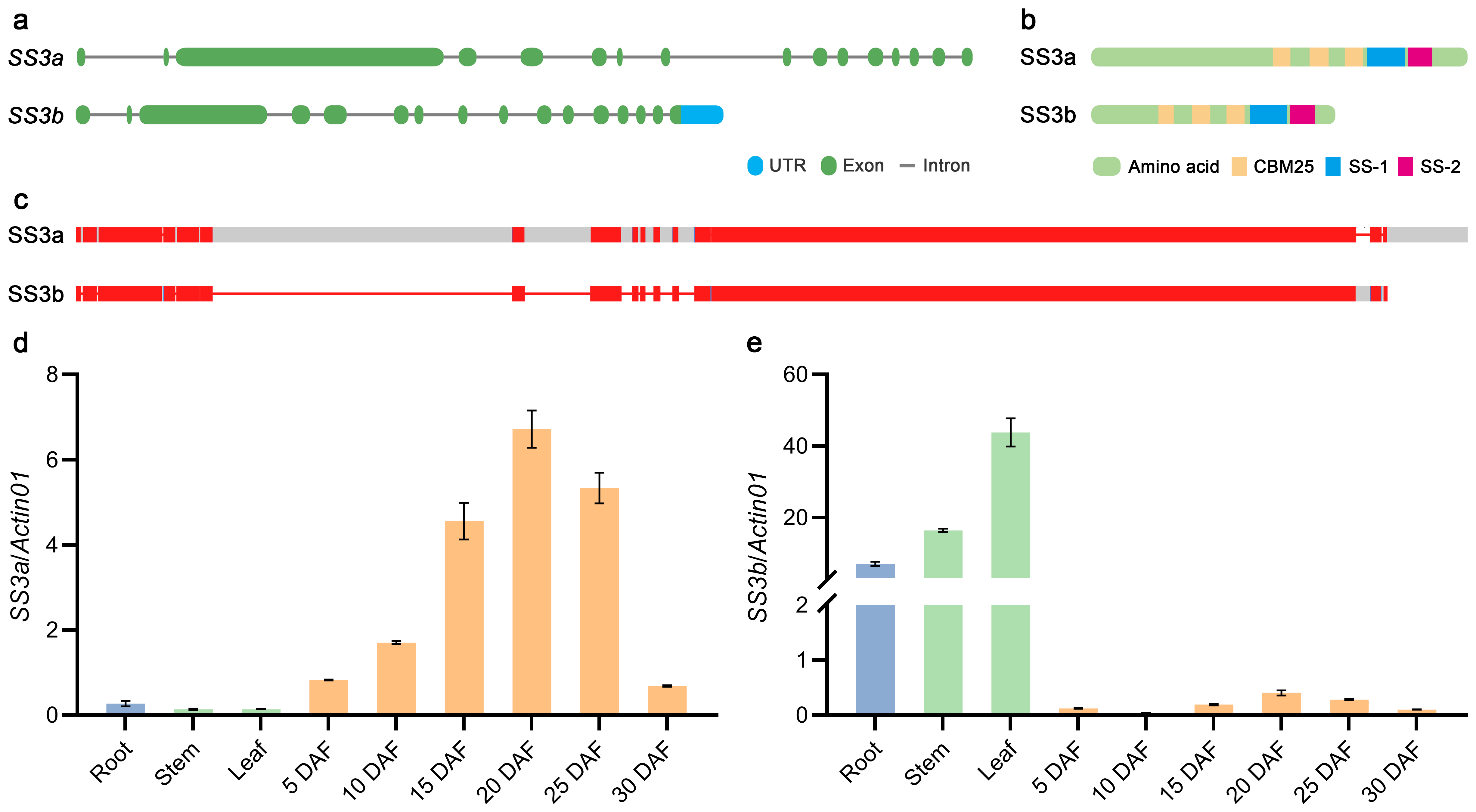
**

**Figure S1. Alignment of the genome and protein sequences between SS3a and SS3b isoforms in rice.** (a-b) Alignment of *SS3a* and *SS3b* gene structures and protein domains. CBM25, Carbohydrate binding module family 25. SS-1 and SS-2, Starch synthase catalytic domain. (c) Alignment of SS3a and SS3b protein sequence conservation. Red indicates highly conserved positions and blue indicates lower conservation. (d-e) Expression patterns of *SS3a* and *SS3b* in rice tissues. DAF, days after flowering.

**
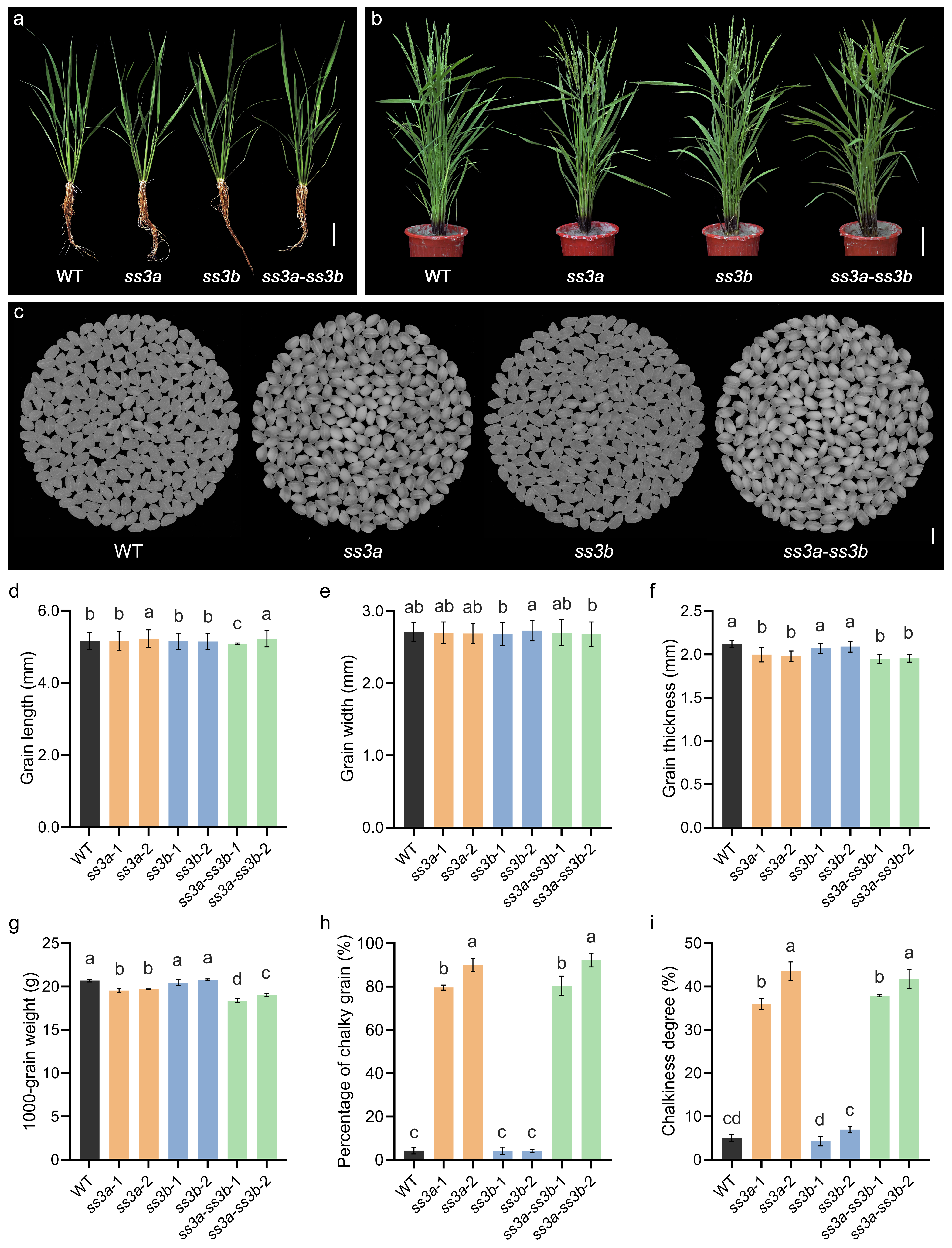
**

**Figure S2. Agronomic performance of *ss3* mutants and their wild type.** (a-b) The plant morphology during vegetative growth and grain filling stage. Scale bar = 5 cm (a), 10 cm (b). (c) The morphology of milled rice. Scale bar = 5 mm. (d-i) The grain length, grain width, grain thickness, 1000-grain weight, percentage of chalky grain and chalkiness degree of brown rice. Different lower-case letters indicate statistically significant differences at *P* < 0.05. The sample size is >100 in (d-e),10 in (f), and 3 in (g-i).

**
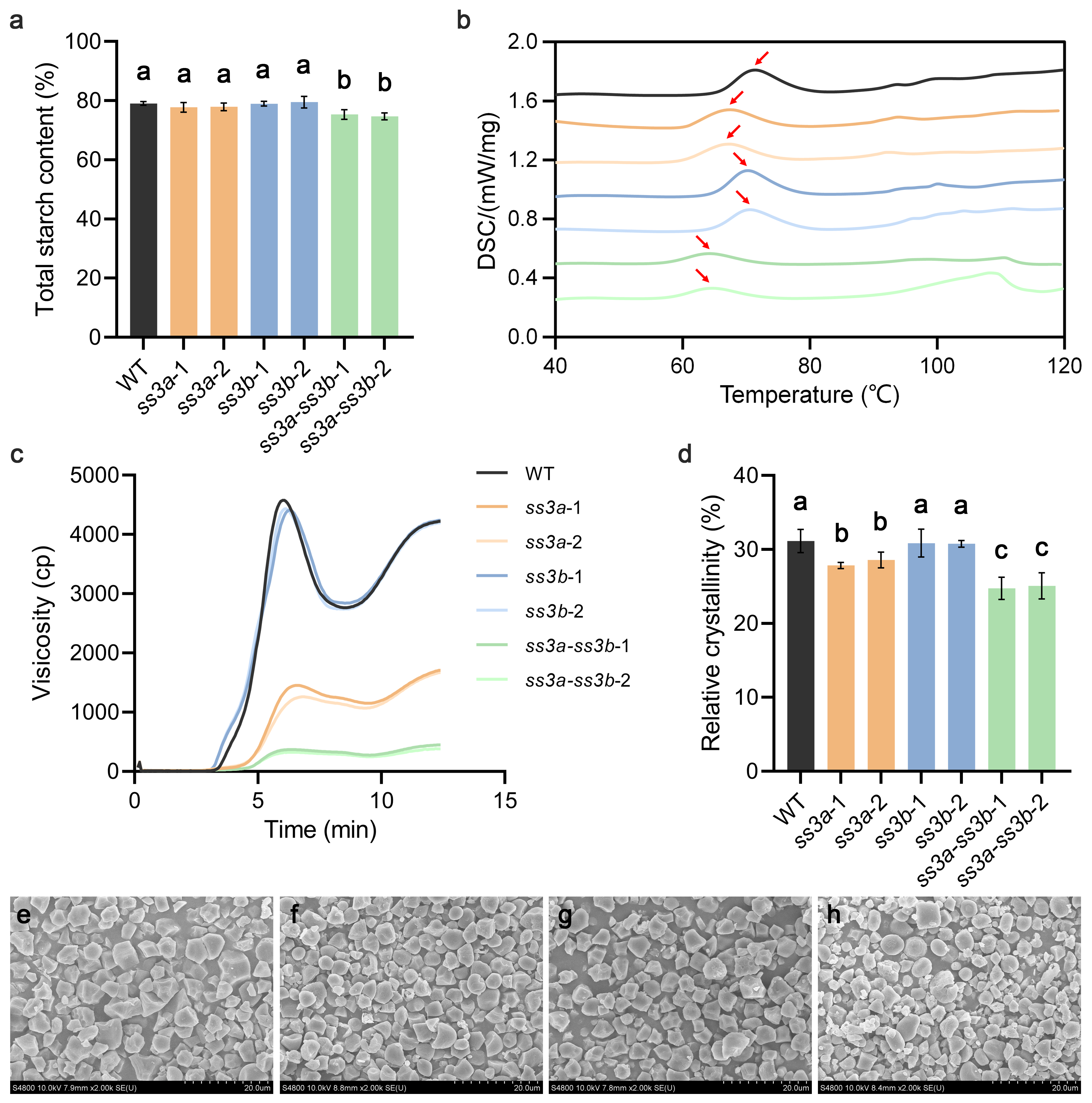
**

**Figure S3. Analysis of physicochemical properties and starch fine structure of *ss3* mutants and their wild type.** (a-c) The total starch content (a) and gelatinization (b) and viscosity (c) properties of rice flour. (d) The relative crystallinity of purified starches. (e-h) The starch granule morphology of purified endosperm starches. From left to right are WT, *ss3a*, *ss3b* and *ss3a*-*ss3b* mutants, respectively. Different lower-case letters indicate statistically significant differences at *P* < 0.05, n=3.

**
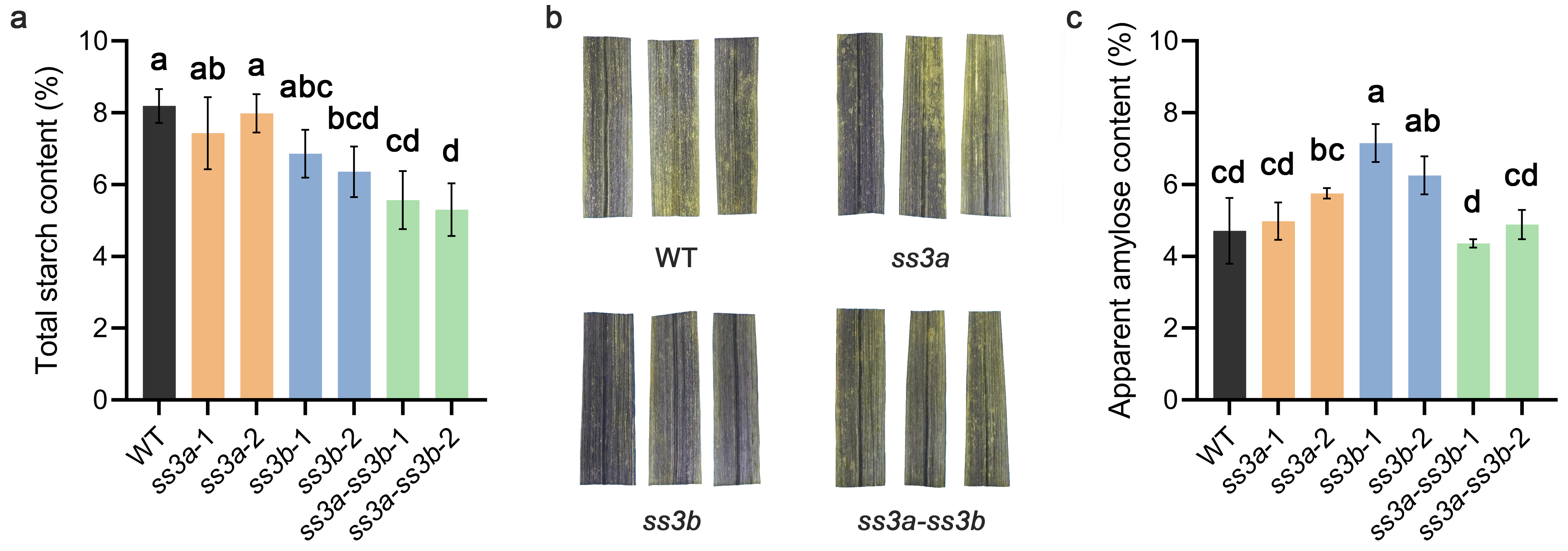
**

**Figure S4. Iodine staining and detection of starch composition in leaves.** (a-c) The total starch content (a), iodine staining (b) and apparent amylose content (c) of leaves. Different lower-case letters indicate statistically significant differences at *P* < 0.05, n=3.

**
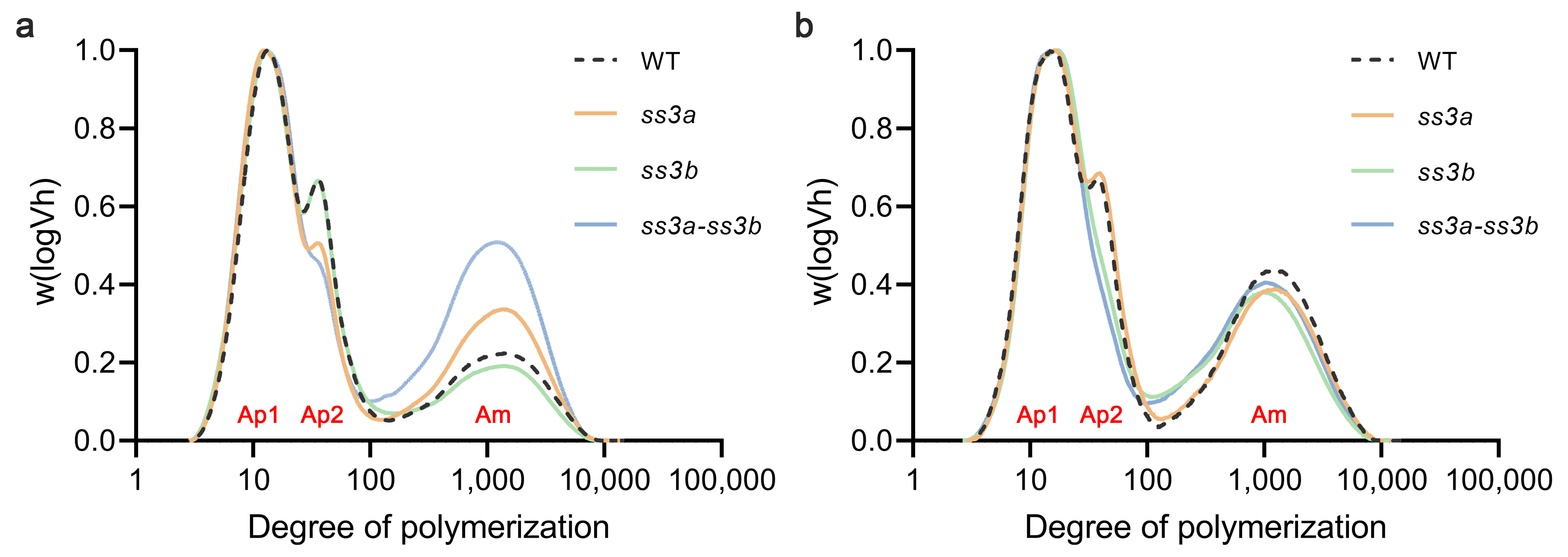
**

**Figure S5. Molecular weight distribution of purified starches from endosperm (a) and leaves (b).** The areas of the three peaks Ap1, Ap2 and Am represent the contents of medium and short chain of amylopectin (Ap1), long chain of amylopectin (Ap2) and amylose (Am), respectively.

**Supplementary Materials and Methods**

**Plant Materials.** The *japonica* rice cultivar Nipponbare (WT) was used to generate *ss3a*, *ss3b* and *ss3a*-*ss3b* mutants via CRISPR/Cas9 technique. All the rice plants were grown under the same climatic and management conditions at the experimental field of Yangzhou University in Yangzhou, China (32°23′N).

**Bioinformatics Analysis.** The gene sequence and structure were downloaded from Gramene website (https://ensembl.gramene.org/Oryza_sativa/Info/Index). The protein domains of SS3a and SS3b was analyzed by InterPro (https://www.ebi.ac.uk/interpro/). The protein sequence of SS3a and SS3b was aligned via the Constraint-based Multiple Alignment Tool (COBALT) (https://www.ncbi.nlm.nih.gov/tools/cobalt/cobalt.cgi?CMD=Web). Coloring method for protein alignment is “Conservation”, which highlights highly conserved and less conserved amino acid positions based on the relative entropy threshold of the residue. Red indicates highly conserved positions and blue indicates lower conservation.

**Vector Constructions and Plant Transformation.** The target sites in *SS3a* and *SS3b* genes (Figure S2A) were designed via the CRISPR-GE (http://skl.scau.edu.cn/) online toolkit and constructed into a CRISPR/Cas9 vector following the described procedure (Wang et al., 2015; Xie et al., 2017). The confirmed construct was introduced into Nipponbare by *Agrobacterium*-mediated transformation. The target DNA region of *SS3a* and *SS3b* were verified by sequencing, and the sequencing data were analyzed by DSDecodeM (http://skl.scau.edu.cn/dsdecode/) or manual decoding (Liu et al., 2015). The homozygous *ss3a*, *ss3b* and *ss3a*-*ss3b* mutants without the T-DNA insertion were screened and selected for following analyses. The primers used are listed in Table S1.

**Measurement of Grain Physicochemical Properties****.** Air-dried mature seeds were dehusked using a SY88-TH instrument (Sangyong, Korea), polished (Kett, Japan), ground into powder (Foss, Sweden) and passed through a 100-mesh screen. The content of total starch and triglyceride of the milled rice ﬂour was analyzed using a total starch assay kit (K-TSTA, Megazyme, Ireland) and a triglyceride content detection kit (BC0625, Solarbio, China), respectively. The apparent amylose content (AAC), gelatinization and viscosity properties were determined according to our previously reported methods (Huang et al., 2021). All tests were performed in duplicate or triple, except for RVA, which is limited by the amount of rice flour.

**Measurement of physicochemical properties of transitory starch in leaves.** Flag leaves at one week after flowering were harvested at the end of the day and ground into powder in liquid nitrogen. After removing the chlorophyll by soaking with 50%, 80% and 100% (v/v) ethanol, part of the leaf powder was freeze-dried and used to determine total starch content using a Megazyme K‐TSTA starch assay kit (Megazyme, Ireland) and AAC using an iodine staining method. The transitory starch was also extracted from the same leaf sample following previously reported method (Wang et al., 2020). The purified starch was used for determination of starch fine structure.

**Measurement of Digestible Characteristics.** 1 g of raw polished rice grain sample were cooked with 1.8 mL deionized water, and then dried in a freeze dryer and ground into powder. The resistant starch (RS) content and total digestible starch content were determined by a digestible and resistant starch assay kit (K-DSTRS, Megazyme, Ireland). The *in vitro* digestion test was analyzed based on the methods described elsewhere (Sopade and Gidley, 2009). The amount digested at different digestion time points (20, 40, 60, 90, 120, 180 min) was calculated from the glucose content.

**Scanning Electron Microscope (SEM).** For SEM observations of the cross cut rice grains, samples were directly mounted on an aluminum stub using carbon double-sided conductive tape. For SEM observations of the starch granules, samples were suspended in ethanol and mounted on an aluminum stub using carbon double-sided conductive tape. The samples were then observed and photographed after being coated with gold using a sputter coater and examined using an environmental scanning electron microscope (Hitachi S-4800II, Japan).

**Size Exclusion Chromatography (SEC) and Fluorophore-Assisted Carbohydrate Electrophoresis (FACE) Analyses.** The grain starch was extracted from milled rice endosperm using the neutral protease method (Wang and Wang, 2001) with slight modifications. Puriﬁed rice grain and leaf starch was debranched with isoamylase (EC3.2.1.68, E-ISAMY, Megazyme), dissolved in DMSO/LiBr, and used to determine the relative molecular weight distribution using a Waters SEC-MALLS system (Wyatt Technology), equipped with differential refractive index (DRI) (Zhu et al., 2020). The three well-resolved fractions from SEC curves represent the content of medium and short chain of amylopectin (Ap1), long chain of amylopectin (Ap2) and amylose (Am), respectively. Debranched starch was also quantitatively analyzed using FACE with a PA-800 Plus instrument (Beckman, USA) and APTS-labelled linear glucans to determine the chain-length distribution (CLD) of amylopectin, denoted *N*de(*X*) (Gu et al., 2019). The Δ*N*de (*X*) value, representing the change in CLD, was then calculated from the *N*de(*X*) value for transgenic lines minus the *N*de(*X*) value for the corresponding WT, where, de indicates linear glucans and X indicates the degree of polymerisation (DP).

**X-Ray Diﬀraction (XRD)**. XRD analysis of the starches was investigated on a D8 Advance X-Ray Diffractometer (Bruker AXS, Germany). All samples were treated in a desiccator with a saturated solution of NaCl to maintain a constant humidity (relative humidity = 75%) for 7 days prior to XRD analysis. The relative crystallinity and amylose-lipid complex content of the starches was determined as previous described (Li et al., 2018; Zhou et al., 2016).

**Statistical Analysis.** Unless otherwise specified, three replicates were performed for each experiment. All data are presented as means ± standard deviations (means ± SD). One-way analysis of variance (ANOVA) was used to determine the level of significance. Different lower-case letters indicate statistically significant differences at *P* < 0.05. “**” indicate significant differences at *P* < 0.01.

**References**

Gu, F., Gong, B., Gilbert, R.G., Yu, W., Li, E., and Li, C. (2019). Relations between changes in starch molecular fine structure and in thermal properties during rice grain storage. *Food Chem.* **295**: 484-492.

Huang, L., Gu, Z., Chen, Z., Yu, J., Chu, R., Tan, H., Zhao, D., Fan, X., Zhang, C., Li, Q., and Liu, Q. (2021). Improving rice eating and cooking quality by coordinated expression of the major starch synthesis-related genes, *SSII* and *Wx*, in endosperm. *Plant Mol. Biol.* **106**: 419-432.

Li, Q.F., Huang, L.C., Chu, R., Li, J., Jiang, M.Y., Zhang, C.Q., Fan, X.L., Yu, H.X., Gu, M.H., and Liu, Q.Q. (2018). Down-regulation of *SSSII-2* gene expression results in novel low-amylose rice with soft, transparent grains. *J. Agr. Food Chem.* **66**: 9750-9760.

Liu, W., Xie, X., Ma, X., Li, J., Chen, J., and Liu, Y. (2015). DSDecode: a web-based tool for decoding of sequencing chromatograms for genotyping of targeted mutations. *Mol. Plant* **8**: 1431-1433.

Sopade, P.A., and Gidley, M.J. (2009). A rapid in-vitro digestibility assay based on glucometry for investigating kinetics of starch digestion. *Starch - Stärke* **61**: 245-255.

Wang, C., Shen, L., Fu, Y., Yan, C., and Wang, K. (2015). A simple CRISPR/Cas9 system for multiplex genome editing in rice. *J. Genet. Genomics* **42**: 703-706.

Wang, L., and Wang, Y.J. (2001). Comparison of protease digestion at neutral pH with alkaline steeping method for rice starch isolation. *Cereal Chem.* **78**: 690-692.

Wang, W., Wei, X., Jiao, G., Chen, W., Wu, Y., Sheng, Z., Hu, S., Xie, L., Wang, J., Tang, S., and Hu, P. (2020). *GBSS-BINDING PROTEIN*, encoding a CBM48 domain-containing protein, affects rice quality and yield. *J. Integr. Plant Biol.* **62**: 948-966.

Xie, X., Ma, X., Zhu, Q., Zeng, D., Li, G., and Liu, Y. (2017). CRISPR-GE: a convenient software toolkit for CRISPR-based genome editing. *Mol. Plant* **10**: 1246-1249.

Zhou, H., Wang, L., Liu, G., Meng, X., Jing, Y., Shu, X., Kong, X., Sun, J., Yu, H., Smith, S.M., Wu, D., and Li, J. (2016). Critical roles of soluble starch synthase SSIIIa and granule-bound starch synthase Waxy in synthesizing resistant starch in rice. *P. Natl. Acad. Sci. U.S.A.* **113**: 12844-12849.

Zhu, J., Yu, W., Zhang, C., Zhu, Y., Xu, J., Li, E., Gilbert, R.G., and Liu, Q. (2020). New insights into amylose and amylopectin biosynthesis in rice endosperm. *Carbohydr. Polym.* **230**: 115656.
